# Epigenetic Stress and Long-Read cDNA Sequencing of Sunflower (*Helianthus annuus* L.) Revealed the Origin of the Plant Retrotranscriptome

**DOI:** 10.3390/plants11243579

**Published:** 2022-12-19

**Authors:** Ilya Kirov, Pavel Merkulov, Ekaterina Polkhovskaya, Zakhar Konstantinov, Mikhail Kazancev, Ksenia Saenko, Alexander Polkhovskiy, Maxim Dudnikov, Tsovinar Garibyan, Yakov Demurin, Alexander Soloviev

**Affiliations:** 1 All-Russia Research Institute of Agricultural Biotechnology, Timiryazevskaya Str. 42, 127550 Moscow, Russia; 2 Moscow Institute of Physics and Technology, 141701 Dolgoprudny, Russia; 3 Federal Research Center of Biological Plant Protection, 350039 Krasnodar, Russia; 4 Skolkovo Institute of Science and Technology, 121205 Moscow, Russia; 5 Pustovoit All-Russia Research Institute of Oilseed Crops, Filatova St. 17, 350038 Krasnodar, Russia

**Keywords:** mobile elements, LTR retrotransposons, Nanopore sequencing, sunflower, epigenetic stress, GAG

## Abstract

Transposable elements (TEs) contribute not only to genome diversity but also to transcriptome diversity in plants. To unravel the sources of LTR retrotransposon (RTE) transcripts in sunflower, we exploited a recently developed transposon activation method (‘TEgenesis’) along with long-read cDNA Nanopore sequencing. This approach allows for the identification of 56 RTE transcripts from different genomic loci including full-length and non-autonomous RTEs. Using the mobilome analysis, we provided a new set of expressed and transpositional active sunflower RTEs for future studies. Among them, a Ty3/Gypsy RTE called SUNTY3 exhibited ongoing transposition activity, as detected by eccDNA analysis. We showed that the sunflower genome contains a diverse set of non-autonomous RTEs encoding a single RTE protein, including the previously described TR-GAG (terminal repeat with the GAG domain) as well as new categories, TR-RT-RH, TR-RH, and TR-INT-RT. Our results demonstrate that 40% of the loci for RTE-related transcripts (nonLTR-RTEs) lack their LTR sequences and resemble conventional eucaryotic genes encoding RTE-related proteins with unknown functions. It was evident based on phylogenetic analysis that three nonLTR-RTEs encode GAG (HadGAG1-3) fused to a host protein. These HadGAG proteins have homologs found in other plant species, potentially indicating GAG domestication. Ultimately, we found that the sunflower retrotranscriptome originated from the transcription of active RTEs, non-autonomous RTEs, and gene-like RTE transcripts, including those encoding domesticated proteins.

## 1. Introduction

Plant genomes possess an enormous number of transposable elements (TEs) and their remnants. TEs are divided into two classes based on their mode of transposition: retrotransposons (class 1) and DNA transposons (class 2). DNA transposons ‘jump’ over the genome via the ‘cut-and-paste’ mechanism. Class 1 TEs mobilize through the reverse transcription of the RNA intermediate molecule and the integration of cDNA into a new genome locus. This mechanism of retrotransposon mobility was discovered in 1985 [1] and is often called ‘copy-and-paste’. Retrotransposons are the most abundant class of TEs in plant genomes [2]. LTR retrotransposons (RTEs) are among the most active TEs in plant genomes. RTEs are divided into two phylogenetically distinct superfamilies: Ty1/Copia and Ty3/Gypsy. The internal part of autonomous RTEs usually carries ORFs encoding the GAG-POL polyprotein [3]. GAG encodes a polyprotein comprising the virus-like particles (VLP) that are needed for the packaging of retrotransposon full-length RNA [4]. The POL polyprotein consists of several subproteins including aspartic protease (AP), reverse transcriptase (RT), RNase H (RH), and integrase (INT), which are involved in the generation of cDNA and its integration into the genome [4]. The transcription of full-length RTE RNA is essential for the RTE life cycle and the generation of cDNA. However, recent RTE transcript analysis using short- as well as long-read sequencing revealed a complex picture of the transcript set expressed from RTEs [5,6,7,8,9]. One common pattern of the RTE transcriptome found in plants including Arabidopsis [5,6,9], triticale [8], and sunflower [7] is the expression of a short isoform, shGAG, encoding the GAG protein. This isoform is expressed at a much higher level then full-length RTE RNA and usually consists of one or a few exons. It was proposed that the overexpression of shGAG enables a molar excess of GAG proteins for correct VLP formation [5,6]. This is also achieved by the higher translation rate of shGAG transcripts and their cytosolic localization [5,6]. As we showed for the sunflower Tyran Ty1/Copia RTE, the transcript set from a single RTE can include multiple isoforms with unknown roles in RTE biogenesis.

In addition to the transcripts expressed from full-length RTEs, a eukaryotic cell may have transcripts expressed from truncated RTEs such as TRIM and TR-GAG elements, as well as RTEs with nested insertions of other elements and RTE remnants possessing LTRs with the promoter activity [10,11,12]. TR-GAG elements include two LTRs flanking the internal part of the encoding GAG protein. TR-GAGs were described in different plant species, and in some of them, they are capable of mobilizing despite being non-autonomous [13,14]. Autonomous and non-autonomous RTEs both significantly contribute to the plant RTE retrotranscriptome. However, there is another source of RTE-related transcripts—domesticated RTE genes. Domesticated RTE proteins have been widely identified in animal genomes (reviewed in [15]). Recently, the global search for domesticated GAG proteins and phylogenetic analysis revealed multiple cases of GAG domestication in plants [16]. The authors showed that one of the methods of GAG protein domestication during evolution is its fusion with a host protein [16]. This evolutionary pattern was also described for transposase domestication in plants and animals [15,17,18]. The function of domesticated GAG proteins in plants is currently unclear, but their fusions with proteins related to plant responses to viruses suggest the potential role of domesticated GAGs in the evolutionary arms race between plants and viruses [16]. It is worth noting that our knowledge about RTE-related transcripts expressed from non-RTE loci is scarce because RTE annotation has been an obligatory step in many pipelines for RTE transcription investigation. Indeed, RTE expression evaluation based on short-read RNAseq data requires prior knowledge about the genomic coordinates of RTEs. In contrast, long-read data are more sufficient for locus-specific RTE expression analysis, as the reads have better mappability to the genome [9]. Additionally, a de novo transcriptome assembly pipeline for long-read cDNA was recently developed. This, together with modern TE annotation tools (e.g., TEsorter [19]) and RTE protein databases (e.g., RExDB [20]), paves the way for the identification of RTE-related transcripts independent of RTE annotation.

Retrotranscriptome analysis is challenging in plants because RTEs are silenced in non-stressed conditions. Methylation is a hallmark of TE sequences in plant genomes, implying an important role of cytosine methylation in TE silencing, with CG methylation playing a major role in suppressing TE expression. This is supported by a number of studies suggesting TE transcription activation in plants with decreased DNA methylation [9,21]. Therefore, different approaches have been used to create TE provocative conditions, including the application of stress [7] or the exploration of plants carrying mutation in key DNA methylation or RNA interference pathways [9]. Recently, Arabidopsis mutants with totally erased cytosine methylation were obtained [21]. These mutants demonstrated TE reactivation, with >1300 TEs upregulated in the mutant plants. In contrast, only 11 new insertions were found in the same plants, suggesting a low correlation between TE transcription and transposition activity. The creation of mutant plants is not an easy task for many plant species, significantly hindering retrotranscriptome and mobilome investigation. To facilitate the investigation of TEs in non-model plants, different chemical inhibitors of DNA methylation were applied, including 5-azacytidine and zebularine [22,23,24]. The treatment of plants with zebularine caused heterochromatin decondensation and diffusion with global cytosine methylation reduction [22]. Zebularine treatment is able to increase or even activate TE transcription [24]. Recently, Thieme et al. [25] proposed a new technique, TEgenesis, for TE mobility activation. The authors showed that the simultaneous application of zebularine and α-amanitin, a PolII inhibitor, resulted in the transgenerational inheritance of new insertions. TEgenesis has been applied to activate TEs in Arabidopsis and rice [25,26].

Here, we combined two powerful approaches, TEgenesis and Oxford Nanopore cDNA sequencing, to uncover retrotranscriptome composition in sunflower and to identify transpositional active TEs. We identified 56 loci of the sunflower genome expressing RTE-related transcripts of different origins. Our mobilome assay provides a new high-confidence set of potentially active sunflower RTEs for future investigation and revealed one Ty3/Gypsy RTE called SUNTY3 that generates eccDNA during epigenetic stress. In addition, our structural analysis demonstrated that the sunflower genome possesses different non-autonomous RTEs that encode a single RTE protein. Finally, we identified nonLTR-RTE transcripts encoding GAG proteins (HadGAG1, HadGAG2, HadGAG3) fused with a host protein, suggesting potential GAG domestication.

## 2. Results

### 2.1. Experiment Design and Oxford Nanopore cDNA Sequencing

**Figure 1:**
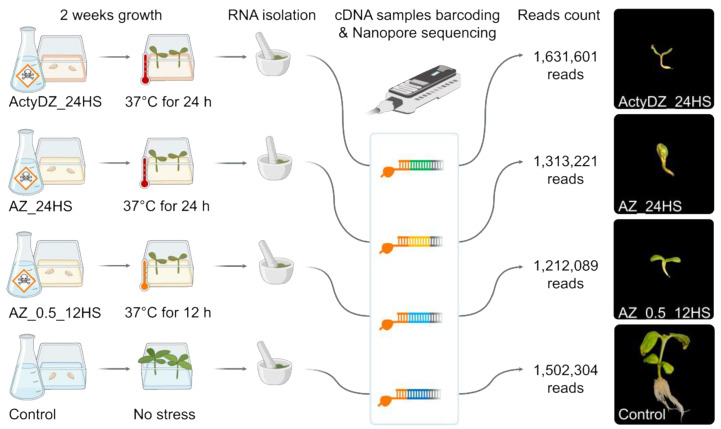
Experimental design of the epigenetic stress of sunflower performed by the heat stress of sunflower seeds germinated in an MS medium containing different concentrations of alpha-amanitin and zebularine toxins or no toxins (Control): AZ_0.5_12HS (2 μg/mL alpha-amanitin, 4 μg/mL zebularine, 12 h of heat stress (37 °C)), AZ_24HS (4 μg/mL alpha-amanitin, 8 μg/mL zebularine, 24 h of heat stress (37 °C), ActyDZ_24HS (4 μg/mL actinomycin D, 8 μg/mL zebularine, 24 h of heat stress (37 °C)). Right panel shows the sunflower plant morphology three weeks after the seeds were placed on MS.

To create epigenetic stress for sunflower, we germinated seeds on an MS medium containing a combination of zebularine (Z) with one of the polymerase II inhibitors, alpha-amanitin (A) or actinomycin D (ActyD) [25]. For the A–Z combination, two concentrations of drugs and heat stress exposure were used to create conventional (AZ_24HS, 4 μg/mL alpha-amanitin, 8 μg/mL zebularine, 24 h of heat stress (37 °C)) and mild stress (AZ_0.5_12HS, 1/2 A–Z concentration, and 12 h of heat stress (37 °C)) conditions (Figure 1). For ActyD-Z treatment (ActyDZ_24HS), 4 μg/mL actinomycin D and 8 μg/mL zebularine in combination with 24 h of heat stress (37 °C) were applied. Plants were grown in the presence of toxins or with no toxins in the medium.

After two weeks, plants grown in the medium containing toxins were obviously different from the control plants. The toxin-treated seedlings showed dwarfism, an impaired development of the stem, and no lateral roots (Figure 1). Additionally, the sizes of the sunflower seedlings and roots were significantly smaller in plants grown on the toxin-containing medium. Unfortunately, plants grown on the toxin-containing medium did not survive after transferring to the soil in the greenhouse.

For Nanopore sequencing, RNA was isolated from plants immediately after the heat stress was finished. For Oxford Nanopore (ONT) cDNA sequencing, the total RNA was converted to cDNA, followed by the barcoding of individual samples. ONT sequencing was run on MinION and resulted in the collection of >1 million reads per sample (Figure 1) with N50 of 1.3 Kb.

### 2.2. LTR Retrotransposon-Related Transcript Detection

**Figure 2:**
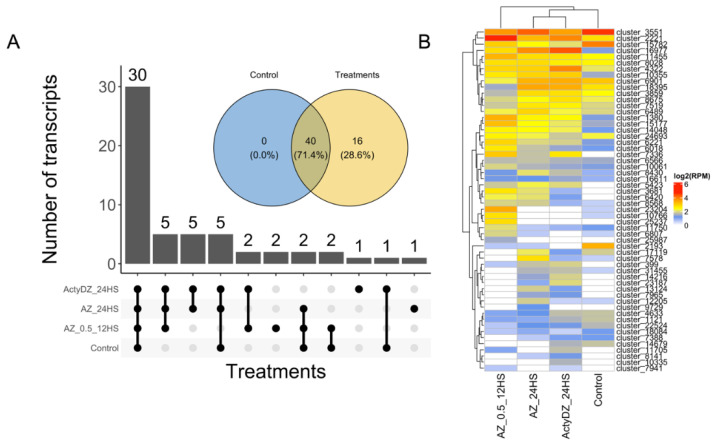
LTR retrotransposon-related transcripts (RTE transcripts) identified by ONT cDNA sequencing. (**A**) Upset plot and Venn diagram showing the number of transcripts detected in different experimental conditions. (**B**) Heatmap of the expression (log2(RPM)) values of RTE transcripts in four experimental conditions: control (normal MS medium without heat stress), AZ_0.5_12HS (2 μg/mL alpha-amanitin, 4 μg/mL zebularin, 12 h of heat stress (37 °C)), AZ_24HS (4 μg/mL alpha-amanitin, 8 μg/mL zebularine, 24 h of heat stress (37 °C), ActyDZ_24HS (4 μg/mL actinomycin D, 8 μg/mL zebularine, 24 h of heat stress (37 °C)).

Taking advantage of ONT cDNA sequencing, we aimed to explore the LTR retrotransposon (RTE)-related transcripts of different origins. To identify RTE-related transcripts, we exploited the following pipeline: (1) the transcripts were de novo assembled by RATTLE [27] using all ONT reads, producing 80,256 consensus sequences; (2) the transcripts were mapped to the genome, and the transcripts mapped with MQ > 30 were kept for further analysis; (3) the assembled transcripts were processed by TEsorter [19], resulting in 151 RTE transcripts; (4) the ONT reads of four samples were mapped to the genome to inspect the read alignment for individual RTE transcripts; (5) the transcripts were manually checked in the genome browser JBrowse2 [28] to assess the quality of the raw ONT read mapping to the RTE transcripts loci; (6) for each RTE transcript, open reading frames (ORFs) were predicted using ORFfinder (https://www.ncbi.nlm.nih.gov/orffinder, accessed on 17 September 2022), and encoding RTE proteins were predicted by the GyDB database [29]. This pipeline led to the identification of 56 high-confidence RTE transcripts (Supplementary Table S1), with 40 RTEs expressed in the control and in any of the treatment samples (Figure 2). Among them, 30 RTE transcripts were detected (>2 RPM, Figure 2B) in all samples, corroborating previous reports, where tens of LTR-RTEs were shown to be expressed in different sunflower tissues under normal conditions [8,30,31].

Meanwhile, 28.6% (16) of the RTE transcripts were not expressed in the control sample and were detected only in samples subjected to TE provocative treatments (Figure 2A,B). This shows that the application of provocative TE conditions may provide an extended list of expressed RTE transcripts for their deeper elucidation.

### 2.3. The Origin of RTE Transcripts

**Figure 3:**
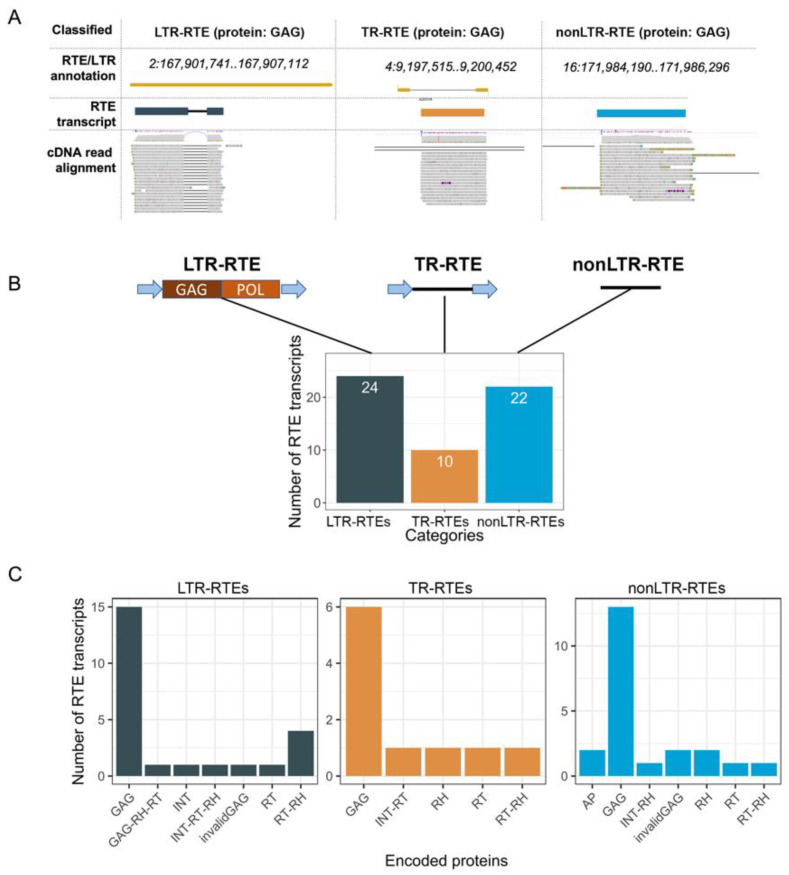
Classification of RTE transcripts into three categories. (**A**) Genome browser snapshots of RTE transcript loci from three categories with the ONT read alignment and LTR-RTE annotation. (**B**) Schematic representation of the structure of the three categories of RTE loci and the number of transcripts for each category. (**C**) Number of RTE transcripts carrying ORFs for different proteins.

To elucidate the origin of RTE transcripts, we classified the RTE transcripts based on their location relative to the LTR retrotransposons that we annotated in the XRQ2.0 sunflower genome using two tools, LTRpred pipeline [32] and LTRharvest [33]. The intersection of the genomic location of the RTE transcript loci and the annotated LTR retrotransposons revealed that 24 RTE transcripts (called LTR-RTEs) were expressed from full-length (22) or truncated LTR retrotransposons (2) (Figure 3). Most of these LTR retrotransposons (21, 87.5%) belong to three lineages (Ale, Angela, and Ivana) of the Ty1/Copia superfamily (Supplementary Figures S1 and S2). The prediction of ORFs and the encoding proteins revealed that 62.5% of the LTR-RTE transcripts carry ORF for the GAG protein. The Ivana lineage differs from the others in terms of encoded proteins because all of its RTE transcripts encode reverse transcriptase (RT) and RNAseH (RH), and no GAG-encoding transcripts were detected. Thus, distinct RTE lineages may differ in a set of ORFs carried by RTE transcripts. Only two of the identified RTEs (cluster_3551 and cluster_6489) have previously been found to also be expressed in sunflower seedlings [7]. One of these RTEs is *Tyran*, a Ty1/Copia RTE with ubiquitous expression in sunflower tissues and organs [7]. Based on this, we may conclude that our current study presents >20 new expressed RTEs of sunflower. It is worth noting that we found no transcripts corresponding to full-length genomic RNA (gRNA) encoding all RTE proteins. This can be explained by the high expression level of the GAG-encoding RNA compared to gRNA [5,6]. Therefore, it may be suggested that the sequencing depth used in our study was too low to detect the gRNA of RTEs.

The next group of RTE transcripts was classified as TR-RTEs (truncated LTR retrotransposons) and included 10 transcripts (Figure 3). They consist of two LTRs and one ORF between them. The TR-RTE ORFs encode one of the following proteins: GAG, RT, RT-RH, RH, and INT-RT. Based on the encoded proteins, TR-RTEs were named as TR-GAG (6), TR-RT (1), TR-RT-RH (1), TR-RH (1), and TR-INT-RT (1) (Supplementary Figures S1 and S2). TR-GAGs have a structural similarity to previously described TR-GAG retrotransposons [7,8,13,14,34,35]. Thus, we showed that the sunflower genome possesses previously unknown non-autonomous LTR retrotransposons carrying ORF for distinct RTE proteins and flanked by two LTRs. The discovered elements significantly extend the classification of plant non-autonomous RTEs.

Surprisingly, we found that 22 RTE-related transcripts have no signature of LTRs. We named these genes-encoding proteins of retrotransposon origin as nonLTR-RTEs. More than half of the nonLTR-RTEs possess an ORF encoding GAG (13 transcripts, 59%). Based on the similarity with known RTE families, we determined that nonLTR-RTEs were derived from 10 RTE lineages: Ale, Alesia, Athila, Ivana, Reina, Retand, SIRE, Tar, Tekay, and Tork. From a phylogenetic point of view, this group of RTE-related transcripts is the most diverged compared to LTR-RTEs and TR-RTEs.

Thus, structural and protein analysis revealed three categories of loci from which RTE-related transcripts are expressed: LTR-RTEs, TR-RTEs, and nonLTR-RTEs. Among them, 34 (60%) transcripts are encoded by potentially autonomous (LTR-RTEs) and non-autonomous (TR-RTEs) RTEs. We may also conclude that 40% of the loci for RTE-related transcripts do not have their LTR sequences, and, therefore, these transcripts (nonLTR-RTEs) resemble conventional eucaryotic genes encoding RTE-related proteins with unknown functions.

To detect any biases in the phylogeny of expressed RTEs, we compared the proportion of RTEs’ different clades in our dataset (LTR-RTEs and TR-RTEs categories) and in the whole genome assembly (Supplementary Figures S3). In concordance with our previous analysis [7], the expressed RTE set was enriched by the Ale clade (20 of 34 vs. 1816 of 23,727, Pearson’s Chi-squared test with Yates’ continuity correction *p*-value < 2.2 × 10^−16^) RTEs. Additionally, members of the largest sunflower RTE clade, Tekay (Ty3/Gypsy), were underrepresented in the expressed RTE dataset compared to the whole genome assembly (4 of 34 vs. 8855 of 23,727, Pearson’s Chi-squared test with Yates’ continuity correction *p*-value = 0.03367).

### 2.4. Transcription Biases of RTE Transcripts

**Figure 4:**
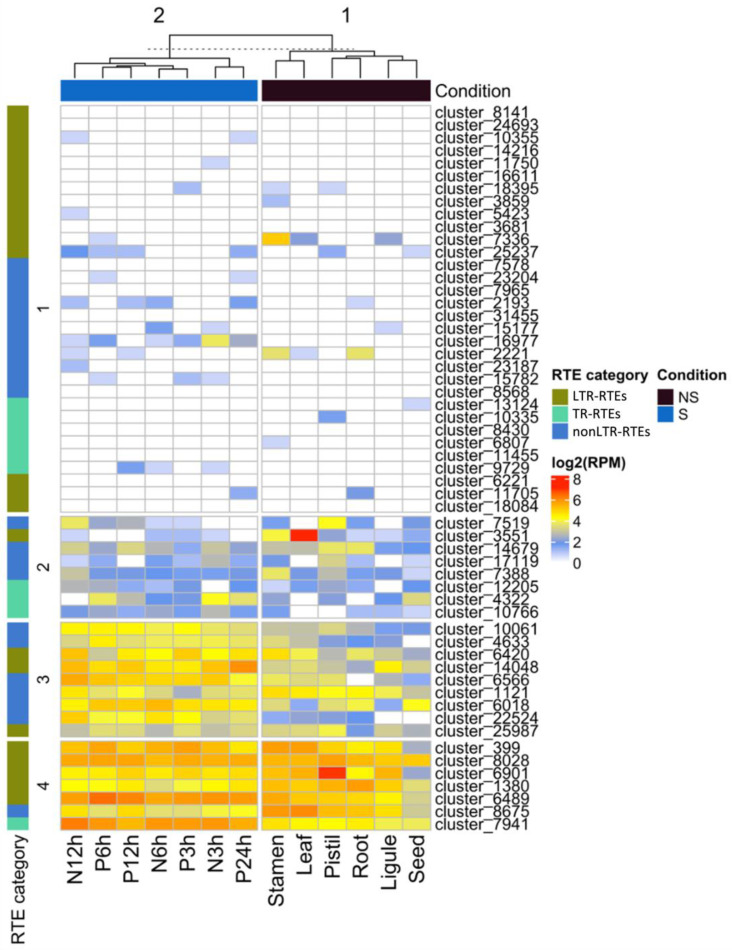
Heatmap of the expression (log2(RPM)) of different categories of RTE transcripts in stress (S) and non-stress (NS) conditions based on RNAseq data. RTE transcripts are clustered based on Pearson distance hierarchical clustering. Clusters 1–4 are labeled on the left side of the heatmap. The materials for the RNAseq used for this analysis were the seed, ligule, root, pistil, leaf, stamen, and plants treated by NaCl (3 (N3h), 6 (N6h), and 12 (N12h) h) and Polyethylene Glycol (3 (P3h), 6 (P6h), 12 (P12h), and 24 (P24h) h).

To understand whether the identified RTEs are specifically expressed in stress conditions and to unravel RTEs with the expression in reproductive organs, we carried out RNAseq analysis. For this, we used publicly available RNAseq data (NCBI project PRJNA483306) from different organs (seed, ligule, root, pistil, leaf, stamen) and stress conditions (NaCl (3, 6, and 12 h) and Polyethylene Glycol (3, 6, 12, and 24 h) treatments). Using Pearson distance hierarchical clustering, we defined four major clusters of RTE transcripts based on their expression patterns (Figure 4). Cluster 1 includes RTE transcripts expressed at a very low level in all samples or without detectable expression. Cluster 2 includes RTE transcripts expressed at a low-middle level in all the samples. Cluster 3 contains RTE transcripts expressed at a middle-high level in all the samples but with a slightly higher expression in stressed samples. Cluster 4 includes RTE transcripts expressed at a middle-high level in all the samples.

We did not detect any significant enrichment of certain RTE transcript categories in any of these clusters. However, we noticed that six out of the nine RTE transcripts of Cluster 3 belong to nonLTR-RTEs.

The expression analysis also revealed that two LTR-RTE (cluster_7336 and cluster_6901) transcripts have their highest expression value in reproductive flower organs, stamens, or pistils. This may indicate that these RTEs have evolved some regulatory sequences to increase the chances for transgenerational inheritance. Additionally, three nonLTR-RTEs transcripts (cluster_2221, cluster_7519, and cluster_7388) have their highest expression value in stamens or pistils. This may suggest the specific function of the encoded proteins during the reproduction process. However, this hypothesis requires further investigation.

Thus, the analysis demonstrated that certain RTE transcripts have a narrow expression in reproductive organs and a higher expression in stressed conditions compared to non-stressed ones.

### 2.5. Transposition Activity of the Expressed RTEs

The application of epigenetic and heat stress may trigger not only the transcription of RTEs but also the transposition [23,25]. To find out which RTEs encoding LTR-RTE transcripts are capable of transposing, we carried out a copy number variation assay. For this, we applied two approaches: (1) the assembly-independent approach, which estimates the variability of normalized RTE coverage by short genomic reads in different sunflower accessions, and (2) the estimation of the number of similar (>80% similarity and >80% coverage) RTE copies in different sunflower assemblies. For these analyses, twenty-four full-length RTEs expressing LTR-RTE transcripts were involved. *Gagarin* [7] transposon was used as the control.

**Figure 5:**
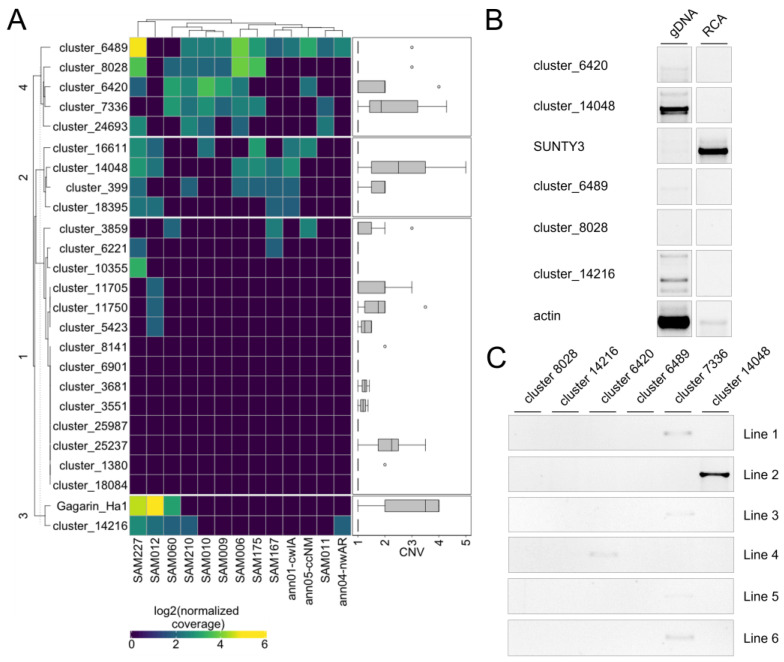
Transposition activity of full-length RTEs expressing LTR-RTE transcripts. (**A**) Heatmap shows the variability of normalized RTE coverage by short genomic reads in different sunflower accessions. The boxplots demonstrate the number of similar (>80% similarity and >80% coverage) RTE copies found in different sunflower genome assemblies normalized by the minimum number of copies among all assemblies. (**B**) EccDNA detection for six selected RTEs (PCR with actin primers is a control on linear DNA removal). (**C**) Inter-retrotransposon amplified polymorphism (IRAP) polymorphism for selected RTEs in six sunflower lines.

Using the first approach, we determined that 16 RTEs (67%) have a detectable increased copy number in at least one accession relative to another (Figure 5A). Of them, 10 RTEs (62.5%) also exhibited an increased copy number in at least one accession based on the assembly-dependent approach (2). These RTEs represent potentially active RTEs of sunflower. We also found that eight (33%) RTEs do not exhibit any significant variation in read coverage among the sunflower accessions. Among them, there was *Tyran* RTE, which we previously showed to be inactive in sunflower leaves despite the high expression level. Three of these RTEs have no copy number variation based on the assembly-dependent approach (2).

To further validate the transposition activity of the RTEs, we selected six RTEs (cluster_6489, cluster_8028, cluster_14216, cluster_6420, cluster_14048, and cluster_7336) and carried out eccDNA (extrachromosomal coiled DNA) analysis in AZ_0.5_12HS plants. EccDNA is a byproduct of the RTE life cycle and can be used as a marker of RTE transposition activity [36]. For this analysis, linear DNA was removed by exonuclease treatment from total genomic DNA, and eccDNAs were amplified by rolling circle amplification (RCA). PCR with primers specifically designed to amplify the LTR–LTR junctions of eccDNA was performed. PCR with five primer pairs did not produce any bands on the RCA product. PCR with an RCA product and cluster_7336 primers resulted in a clear PCR product (Figure 5B), demonstrating the ongoing transposition activity of this RTE. RTE cluster_7336 is the Ty3/Gypsy retrotransposon and was renamed to SUNTY3. We also performed IRAP analysis using the same primer pairs and 15 sunflower lines of VNIIMK collection. PCR with primers on SUNTY3, cluster_6420, and cluster_14048 resulted in the amplification of PCR products in 4 lines, 1 line, and 1 line, respectively (Figure 5C). The observed IRAP polymorphism indicates that the RTEs are inserted in tandem order in the sunflower lines and suggests the recent transposition activity of the RTEs.

To conclude, our mobilome analysis revealed 10 RTEs that are expressed and exhibit copy number variation based on the assembly-independent and assembly-dependent approaches. Eleven RTEs demonstrated copy number variation based on one of these approaches. One identified RTE, SUNTY3, is capable of generating eccDNA under epigenetic stress, and three tested RTEs have recent transposition activity detected by the IRAP polymorphism.

### 2.6. nonLTR-RTEs Included Domesticated GAG Genes

**Figure 6:**
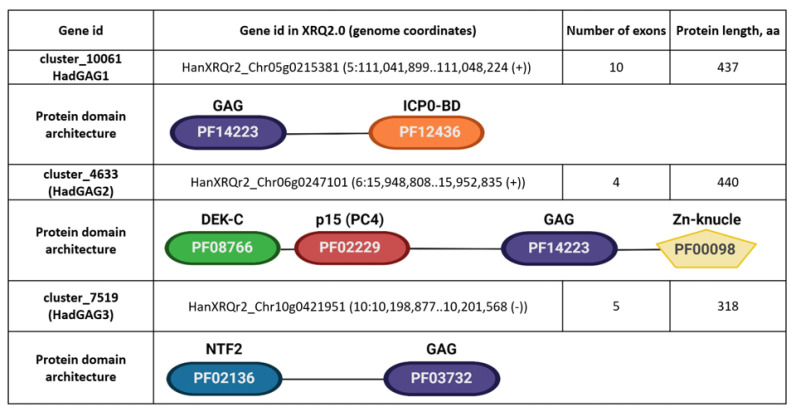
Genome location and domain architecture of nonLTR-RTEs encoding HadGAG proteins.

We noticed that three nonLTR-RTEs genes, cluster_10061, cluster_4633, and cluster_7519 (Figure 6), encoding proteins with a similarity to GAG proteins have only a partial similarity to GAG. Moreover, these nonLTR-RTEs genes have a high exon number (4–10 exons, Figure 6) compared to other GAG-encoding loci (1–3 exons). This motivated us to further elucidate these genes.

Blastp search revealed HadGAG1 homologs in *Artemisia annua* (PWA43159.1, renamed to AanGAG1) and *Tanacetum cinerariifolium* (GEW58681.1, renamed to TciGAG1). Both species belong to the *Asteraceae* family. An analysis of the protein domain architecture via the GenomeNet MOTIF finder showed that the HadGAG1 protein possesses two major protein domains, PF14223 (139 aa-long Copia-type GAG polypeptide) and PF12436 (181 aa-long ICP0-binding domain of Ubiquitin-specific protease 7). AanGAG1 and TciGAG1 shared the same protein domains with HadGAG1, as well as several additional ones. AanGAG1 contained PF14533 (USP7 C-terminal domain), and TciGAG1 contained PF00917 (MATH domain) and PF13976 (GAG-pre-integrase domain). Based on these data, we performed a protein domain architecture-associated search in the InterPro database using HadGAG1 architecture as a query. This search helped us to find another homologous HadGAG1 protein in *Rosa chinensis* (A0A2P6QK79_ROSCH, further RchGAG1). Despite the presence of additional protein domains, PF14223 and PF12436 seem to be the core ones found in the proteins of the *Asteraceae* and *Rosaceae* families. According to TimeTree (TimeTree.org), these families have a common ancestor, 112–125 MYA. The identified protein domains suggest that a function of HadGAG1 (and its homologs) may be associated with the ubiquitinoylation process.

**Figure 7:**
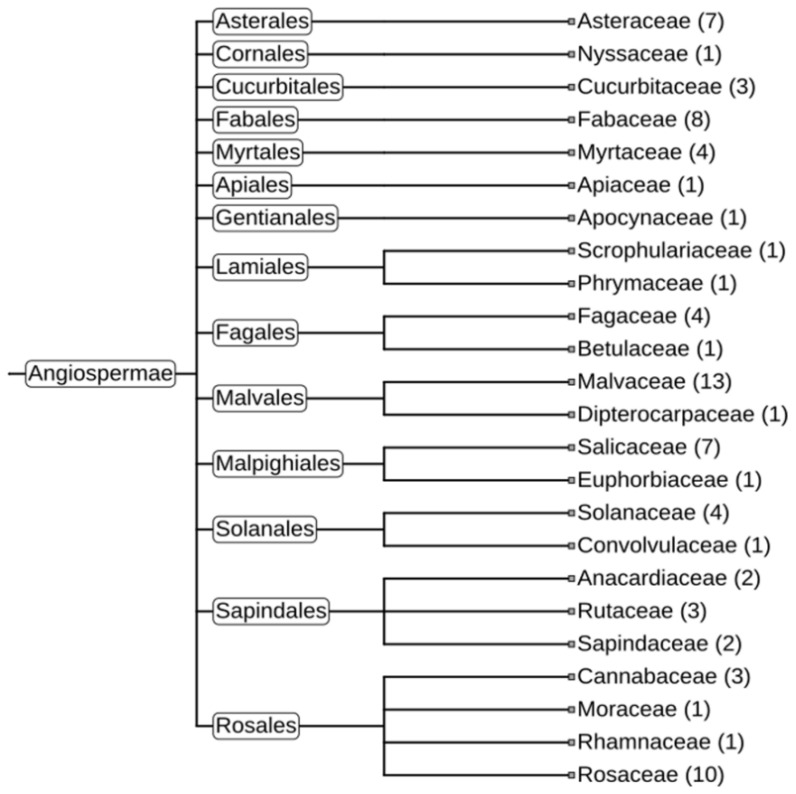
Phylogeny tree of plants containing HadGAG2 homolog proteins (in brackets—number of plant species where HadGAG2 homologs were found). The tree was constructed using iTol [37].

A similar set of analyses was performed with the other two gene clusters, cluster_4633 (HadGAG2) and cluster_7519 (HadGAG3). The HadGAG2 protein exhibited a strong similarity to the Ctrg10 protein, which was recently shown to originate via GAG domestication in plants (Wang and Han 2021). The comparison of the HadGAG2 protein sequence with the sequences of other proteins using BLASTp showed a high level of similarity with homologous proteins from numerous plant species belonging to 24 plant families (Figure 7, Supplementary Figures S4 and S5).

HadGAG2 homolog proteins possess four major protein domains: PF14223 (Copia-type GAG polypeptide), PF08766 (DEK C-terminal domain), PF02229 (Transcriptional Coactivator p15 (PC4)), and PF00098 (Zinc knuckle). A domain architecture-associated search revealed four domain architectures. The differences between these architectures are due to the different numbers of zinc knuckle domains (one, two, five, and six, respectively). Protein homologs containing five and six zinc knuckle domains which can be found in *Nicotiana benthamiana* are of particular interest, since such multiplication of these domains is not typical for retroelements (Klein et al. 2000) and may indicate the further evolution of HadGAG2 in this species.

HadGAG3 blastp analysis showed only one potential homologous protein in *Ambrosia artemisiifolia* (KAI7750139.1, further AarGAG3) from the *Asteraceae* family. These proteins share a high level of protein sequence similarity as well as a protein domain structure. Both of them contain PF03732 (retrotransposon gag protein) and PF02136 (nuclear transport factor 2 (NTF2) domain) with a nuclear localization signal, as confirmed by LOCALIZER and DeepLoc tools.

## 3. Discussion

Here, we combined two powerful approaches, TEgenesis and Oxford Nanopore cDNA sequencing, to uncover the retrotranscriptome composition in sunflower. We identified 56 loci of the sunflower genome expressing RTE-related transcripts. These transcripts fell into one of three categories: LTR-RTEs (transcripts expressed from autonomous RTEs), TR-RTEs (transcripts expressed from non-autonomous RTEs), and nonLTR-RTEs (transcripts expressed from loci that are not flanked by LTRs). The latter category included putative domesticated GAG proteins fused with a host protein. Our mobilome analysis revealed ten RTEs that are expressed and exhibit copy number variation based on assembly-independent and assembly-dependent approaches. Eleven RTEs demonstrated copy number variation based on one of these approaches. At the same time, three RTEs were classified as inactive based on both approaches. One newly discovered RTE that we called SUNTY3 also showed ongoing activity generating eccDNA after epigenetic stress. Our study provides a new high-confidence set of potentially active sunflower RTEs for future investigations.

The application of Nanopore sequencing to detect TE expression has many advantages, including the ability to detect full-length TE transcripts, the high mappability and specificity rate of the reads [9], the simultaneous sequencing of several samples using barcoding, and the identification of individual TE isoforms encoding distinct proteins. It is worth noting that the ONT reads used in this study were generated on the R9.4.1 flow cell, which resulted in a substantial number of errors. Oxford Nanopore sequencing is a quickly evolving technology, and the recent R10.3 and R10.4 flow cells in combination with Q20 chemistry and the modern basecaller (e.g., ‘Bonito’) allow for a significant decrease in the error rate [38], although the read yield is lower compared to that of the R9.4.1 flow cell [39]. Additionally, the exploitation of PacBio HiFi cDNA reads combining low error rates and a relatively long length is another attractive alternative for retrotranscriptome characterization in plants [9]. 

We established the expression of full-length RTEs as well as truncated RTEs possessing two LTRs. The identified truncated RTEs include not only previously characterized TR-GAG non-autonomous RTEs [7,8,13,34,35] but also other TR-types: TR-RT, TR-RT-RH, TR-RH, and TR-INT-RT. Thus, we showed that the sunflower genome possesses previously unknown non-autonomous LTR retrotransposons carrying ORF for distinct RTE proteins and flanked by two LTRs. The discovered elements significantly extend the classification of plant non-autonomous RTEs and pose a question about the roles of these elements in the mobilome or host biology. It is worth noting that previous studies of RTE transcription in plants often used RTE annotation files for short- or long-read assignments [8,9,27,28,37]. In this approach, RTEs that are not properly annotated or RTE-derived sequences lacking the structural features required for annotation are not involved in the expression analysis. Here, for the identification of RTE-related transcripts, we did not focus our analysis only on annotated RTEs but rather used the de novo transcriptome assembly. This allows us to show that the full-length RTEs or truncated RTEs produced only 60% of all the RTE-related transcripts detected in the sunflower. Meanwhile, 40% of the RTE transcripts were expressed from loci that are not flanked by LTRs (nonLTR-RTEs). We anticipate that some of these may be the result of errors in the genome assembly. However, we identified three nonLTR-RTEs that encode GAG proteins which (1) are fused with one of the host proteins, (2) share their homology with another plant species on a protein domain level, and (3) possess 4–10 exons. These features indicate that HadGAG genes may have originated from GAG protein domestication. GAG domestication events have been described in humans, animals, and insects (as reviewed in [12]), while domesticated GAGs were only recently described in plants [13]. One of the HadGAG genes, HadGAG2, was also described by Wang and Han as Ctrg10, a fusion protein between GAG and the transcription coactivator KELP. It was proposed that Ctrg10 is involved in the evolutionary arms race between hosts and viruses. Other HadGAG genes—HadGAG1 and HadGAG3—are described here for the first time. They represent the protein fusion of GAG with the ubiquitin-specific protease 7 C-terminal domain and nuclear transport factor 2 (NTF2) domain, respectively. These two proteins have fewer homologous sequences in other plant species than HadGAG2, suggesting their recent co-option or convergent evolution.

While the plant genome may consist of millions of TE copies and their remnants, only a small portion of them have ongoing transposition activity. This group of TEs is fascinating, as it allows us to study mobilome biology after the activation of these TEs. Although enormous progress has been achieved in the investigation of TEs for model plants [38,39,40,41,42], the progress in the study of TE biology for crops is still lagging behind. One of the reasons for this is the complex genome with error-prone genome assembly that is available. This problem is state-of-the-art, even for the small Arabidopsis genome, where tens of TE copies are not located in the genome assembly. To enable a TE to enter its life cycle, the TE needs to be activated. The utility of TE transposition activation by A–Z treatment is an attractive technique for mobilome investigation in recalcitrant plants for which obtaining stable DNA methylation mutants is often challenging. The application of TEgenesis to sunflower allowed us to capture transpositionally active RTEs. One of these RTEs, which we called SUNTY3, belonged to the Galadriel lineage of the Ty3/Gypsy RTE superfamily. We detected the expression of SUNTY3 only in plants growing on toxins and which underwent heat stress. SUNTY3 expression was also identified in sunflower stamens using RNAseq data. While we have not checked this in our work, it is intriguing to analyze the transposition activity of SUNTY3 in stamens. In general, there are not many examples of plant Ty3/Gypsy RTEs with ongoing transposition activity [33]. Therefore, SUNTY3 can be a new model for the elucidation of plant Ty3/Gypsy biology. In addition, the understanding of which TEs are activated after Tegenesis may facilitate the subsequent analysis of progeny from M1 plants using available methods such as TE-capture [38] and CANS [23]. CANS is a very sensitive method, and it allows for the detection of both somatic and genetically inherited insertions. Therefore, it can be used to perform a bulk analysis of plants with subsequent PCR screening to identify plants with target insertions.

Unfortunately, we did not succeed in growing sunflower plants after the TEgenesis protocol. The application of actinomycin D instead of alpha-amanitin, as well as the decreasing toxin concentration and heat stress duration, did not help to overcome the problem. Probably, the zebularine concentration is more critical for the plant survival rate than the alpha-amanitin concentration and heat stress duration. Previous studies on the application of zebularine to decrease DNA methylation also showed that plants grown on zebularine medium had abnormal development, including the absence of lateral roots and small plant sizes [19,20,43]. Future studies are required to optimize TEgenesis for sunflower and other plant species to manage the plant mobilome and to induce epigenetic changes for plant breeding purposes.

## 4. Materials and Methods

### 4.1. Plant Material

Sunflower seeds of VIR769, BC4 ANN 2138, K2257- 3, BK464, K2058, K1587, RIL29, VK905 A, VK1klpA, VK876A, LG26, VK1-imiA, K578, ZS, and VF lines were provided by the Pustovoit All-Russia Research Institute of Oilseed Crops (Moscow, Russia). 

### 4.2. Toxin Treatment of Sunflower Plants

The toxin treatment for TE activation has been performed as previously described [22]. For aseptic growth, seeds of the sunflower line ‘ZS’ were sterilized in 10% bleach for 10 min, rinsed three times with sterile H2O, and sown on 9 cm Petri dishes containing MS media (1% sucrose, 0.6% agar (Dia-M, 3440.0500), pH 5.7) supplemented with a combination of sterile filtered 5 µM α-amanitin (dissolved in H2O) or actinomycin D (4 μg/mL) and 40 µM zebularine (dissolved in DMSO) [22]. Plants were grown on the toxin-containing medium (or the medium without toxins) for 15 days. Then, the plants were placed at 4 °C in the dark for 1 day, followed by 24 h of heat stress at 37 °C (HS). 

### 4.3. DNA Isolation

DNA was isolated by the cetyltrimethylammonium bromide (CTAB) protocol [44].

### 4.4. RNA Isolation and cDNA Sequencing

Sunflower seeds of this line were germinated at room temperature on wet filter paper disks. Total RNA was isolated from 50 mg material that was homogenized in liquid nitrogen by the ExtractRNA kit (Evrogen, Moscow, Russia) following the manufacturer’s instructions. The RNA concentration and the quality of the isolated RNA were estimated by Nanodrop (Nanodrop Technologies, Wilmington, CA, USA) and gel electrophoresis using 1.2% agarose gel with ethidium bromide staining. The results were detected using a Gel Doc XR+ UV camera (Bio-Rad, Hercules, CA, USA). One microgram of the extracted RNA was DNase digested for 60 min at 37 °C in a final volume of 10 μL containing 0.5 μL of DNase I and 1 µL of RDD Buffer (Qiagen, Germantown, MD, USA) and incubated for 10 min at 75 °C with the addition of 1 µL EDTA of 25 μM EDTA.

A total of 2 µg of DNase-treated total RNA was used for reverse transcription. In MmLV-catalyzed reactions, RNA was primed with poly-A-specific hexamers from the MINT cDNA kit (Evrogen, Moscow, Russia), according to the manufacturer’s instructions. During the synthesis, the optimal number of PCR cycles (20 cycles) was adapted to reach the exponential phase of amplification. The ds-cDNA concentration and quality were estimated by Nanodrop (Nanodrop Technologies, Wilmington, CA, USA) and by Qubit (Qubit dsDNA BR Assay Kits, ThermoFisher Scientific, Waltham, MA, USA) and were checked by gel electrophoresis. Synthesized ds-cDNA was purified by 1.8x Agencourt AMPure XP Beads (Beckman Coulter, Pasadena, CA, USA) in accordance with the manufacturer’s instructions.

For Nanopore sequencing, a library was prepared from ds-cDNA using the nanopore native barcoding genomic DNA SQK-NBD110-24 (Oxford Nanopore Technologies, Oxford, UK), with some modification in the process of using the NEBNext Companion Module for Oxford Nanopore Technologies Ligation Sequencing (New England Biolabs, MA, USA). Roughly 500 ng of each ds-cDNA in 24 µL was mixed with 1.75 µL of NEBNext FFPE DNA Repair Buffer, 1.75 µL of Ultra II End-prep reaction buffer, 1.5 µL of Ultra II End-prep enzyme mix, and 1 µL of NEBNext FFPE DNA Repair Mix and was incubated at the thermal cycler for 5 min at 20 °C and for 5 min at 65 °C. The DNA samples were transferred to a new tube, and an extra 30 µL of H2O was added to reach the required volume for the next preparations of the DNA library. Each end-prepped sample was barcoded with 2.5 µL native barcodes from the SQK-NBD110-24 kit (Oxford Nanopore Technologies, Oxford, UK). After purification, four barcoded DNAs were combined to a final volume of 65 µL, with the further ligation of adapters for sequencing using Quick T4 DNA ligase (New England Biolabs, MA, USA) according to the SQK-LSK109 kit (Oxford Nanopore Technologies, Oxford, UK). 

Sequencing was performed by MinION equipped with the R9.4.1 flow cell. The sequencing process was controlled by MinKNOW software v19.12.5 (Oxford Nanopore Technologies, Oxford, UK). Basecalling was carried out by Guppy v5.0.14 5 (Oxford Nanopore Technologies, Oxford, UK).

### 4.5. eccDNA Isolation and Amplification

Extrachromosomal circular DNA (eccDNA) was isolated from 3 µg of genomic DNA and amplified according to the previously described protocol [40], with several modifications [8]. For double-stranded linear DNA digestion, genomic DNA was treated with Plasmid-Safe ATP-Dependent DNAse (Epicenter, Madison, WI, USA) for 120 h at 37 °C, with an extra amount of reagents added (10 units of Plasmid-Safe ATP-Dependent DNase; 2 µL 25 mM ATP; 0.3 µL Plasmid-Safe 10x Reaction Buffer) after 48 and 96 h. DNA precipitation was carried out by adding 0.1 volume 3 M sodium acetate (pH = 5.2) and 2.5 volume absolute ethanol, followed by overnight incubation at −20 °C. Precipitated eccDNA was amplified by random rolling circle amplification (RCA) reaction using the Illustra TempliPhi 100 Amplification Kit (GE Healthcare, Chicago, IL, USA) for 65 h at 28 °C. RCA reaction products were diluted five times and exposed to inverse PCR with the RTE-specific primers listed in Table 1. 

The PCR conditions were: 94 °C for 1 min; 35 cycles of 94 °C for 1 min, 58 °C for 1 min, and 72 °C for 1 min; and a final elongation at 72 °C for 3 min. 

### 4.6. IRAP

For the IRAP polymorphism analysis, DNA from 15 sunflower lines (VIR769, BC4 ANN 2138, K2257-3, BK464, K2058, K1587, RIL29, VK905 A, VK1klpA, VK876A, LG26, VK1-imiA, K578, ZS, VF) was used. The DNA was isolated according to the protocol [44]. PCR was performed with the ScreenMix kit (Evrogen, Moscow, Russia) according to the manufacturer’s instruction. The PCR program for IRAP was as follows: 95 °C—3 min, 35 cycles (95 °C—30 s, 59 °C—30 s, 72 °C—40 s), 72 °C—3 min. The primers used for IRAP are listed in Table 1. 

### 4.7. Bioinformatic Analysis of RTE Transposition Activity

The assembly-independent approach for the estimation of the variability of the normalized RTE coverage by short genomic reads in different sunflower accessions was performed by the RepeatProfiler pipeline [45], as described previously [7]. For the genome assembly-based approach, the genomes of the eight sunflower lines were downloaded from https://www.heliagene.org/index/Huang_et_al_2022.html (accessed on 11 September 2022). The sequences of 24 RTEs were blasted against the assemblies, and hits with >80% similarity and >80% coverage of the RTE query were kept for further analysis. The heatmap was generated using the ComplexHeatmap R package [46].

### 4.8. RTE-Related Transcript Identification Pipeline

The pipeline for the identification of RTE-related transcripts included several steps. ONT cDNA reads were mapped to the XRQ2.0 sunflower genome [47] using minimap2 [48] with the following parameter: ‘-ax splice’. The obtained sam files were converted to a bam file and sorted using samtools [49]. The bam files were added to the locally installed JBrowse2 [25] for RTE transcript manual verification. For de novo transcriptome assembly, all cDNA reads were combined into one fastq file, and RATTLE software (‘rattle cluster’ and ’rattle correct’) [24] was applied. TEsorter [16], with the assembled transcripts, was used to keep only RTE-related transcripts and to predict encoded RTE proteins. The candidate RTE-related transcripts were mapped to the genome using minimap2 [48]. To find the RTE-related transcripts, the RTEs were predicted in the XRQ2.0 genome using two software: LTRpred [29] and LTRharvest [30].

### 4.9. Domesticated GAG (HadGAG) Gene Analysis

HadGAG1, HadGAG2, and HadGAG3 protein amino acid sequences were used for the possible protein homologs search via the NCBI blastp tool (https://blast.ncbi.nlm.nih.gov/Blast.cgi, accessed on 15 November 2022). Raw data for the phylogeny tree building were obtained from the NCBI Taxonomy Browser (https://www.ncbi.nlm.nih.gov/Taxonomy/taxonomyhome.html/index.cgi, accessed on 15 November 2022). The approximate time period of the evolutionary plant species division was evaluated via the TimeTree tool (http://www.timetree.org/, accessed on 15 November 2022). This tool was also used for building phylogenetic trees. Raw data from the NCBI Taxonomy Browser was processed via the iTol tool (https://itol.embl.de, accessed on 15 November 2022). For the analysis of possible protein intracellular localization, we used several tools: LOCALIZER (https://localizer.csiro.au, accessed on 14 November 2022), TargetP (https://services.healthtech.dtu.dk/service.php?TargetP-2.0, accessed on 14 November 2022), and DeepLoc (https://services.healthtech.dtu.dk/service.php?DeepLoc-2.0, accessed on 14 November 2022). 

### 4.10. RNAseq Analysis of RTE Expression

The RNA-seq data for RTE expression analysis were retrieved from NCBI SRA data (Bioproject: PRJNA483306). The reads were mapped to the XRQ2.0 sunflower genome using Hisat2 [50], followed by bam file generation and sorting by samtools [49]. The read count was performed using featureCounts [51] using the following argument set: -T 64 -C -Q 10 –ignoreDup. The heatmap was generated using the ComplexHeatmap R package [46].

### 4.11. Statistical Analysis and Visualization

Statistical analysis was carried out in Rstudio Version 2021.09.1 (http://www.rstudio.com/, accessed on 16 November 2022) with R version 4.2.0. Bar plots and upset plots were drawn by ggplot2 [52]. Heatmaps were constructed by the ComplexHeatmap [46] R package.

## Figures and Tables

**Table 1:** Primers used for eccDNA detection.

RTE ID	Primers (F and R)
cluster_6489	GAACCCAGAAATCGCACAATC; TGGATGTGTATCAACATGACTCC
cluster_8028	TAGTGTACTGTGAGAGTGAGAAGC; CCAACAGGTGGTTCCTCAAAC
cluster_14216	TGGTTCCATTTGGGTCTTCC; CATGCGGCAGCCGAAATTAT
cluster_6420	GAACAGAGGAATGCCCAGAAA; ACTTCTGGTAAACCAACATGAGA
cluster_14048	ATTTGGCTTCTCCACAGCAG; ATCGTCCCACCATACTTTGTG
cluster_7336	CGAGGACGTCGACAGATTAG; TCATCGCGGTCTCTAGTTCA

## Data Availability

The nanopore data produced for this study are available in the Sequence Read Archive (SRA) NCBI under Bioproject Accession PRJNA910316.

## Supplementary Materials

The following supporting information can be downloaded at: https://www.mdpi.com/article/10.3390/plants11243579/s1, Table S1: Genomic features and encoded retrotransposon domains of 56 expressed RTEs; Figure S1: Bar plot showing the number of RTE transcripts of different categories expressed from Ty1/Copia and Ty3/Gypsy; Figure S2: Bar plot showing the number of RTE transcripts of different categories expressed from distinct lineages of Ty1/Copia and Ty3/Gypsy; Supplementary Figure S3: Bar plot showing the number of RTEs of different categories expressed from distinct lineages of LTR retrotransposons; Figure S4: Phylogenetic tree of species containing HadGAG2 homologs; Figure S5. Phylogenetic tree based on the blastp hits sequences alignment of HadGAG2 homologs.

## References

[1] Boeke J.D., Garfinkel D.J., Styles C.A., Fink G.R. (1985). Ty elements transpose through an RNA intermediate. Cell.

[2] Feschotte C.E.D., Jiang N., Wessler S.R. (2002). Plant transposable elements: Where genetics meets genomics. Nat. Rev. Genet..

[3] Bennetzen J.L., Wang H. (2014). The Contributions of Transposable Elements to the Structure, Function, and Evolution of Plant Genomes. Annu. Rev. Plant Biol..

[4] Sabot F., Schulman A.H. (2006). Parasitism and the retrotransposon life cycle in plants: A hitchhiker’s guide to the genome. Heredity.

[5] Oberlin S., Sarazin A., Chevalier C., Voinnet O., Marí-Ordóñez A. (2017). A genome-wide transcriptome and translatome analysis of *Arabidopsis* transposons identifies a unique and conserved genome expression strategy for *Ty1/Copia* retroelements. Genome Res..

[6] Oberlin S., Rajeswaran R., Trasser M., Barragán-Borrero V., A Schon M., Plotnikova A., Loncsek L., Nodine M.D., Marí-Ordóñez A., Voinnet O. (2021). Innate, translation-dependent silencing of an invasive transposon in *Arabidopsis*. EMBO Rep..

[7] Kirov I., Omarov M., Merkulov P., Dudnikov M., Gvaramiya S., Kolganova E., Komakhin R., Karlov G., Soloviev A. (2020). Genomic and Transcriptomic Survey Provides New Insight into the Organization and Transposition Activity of Highly Expressed LTR Retrotransposons of Sunflower (*Helianthus annuus* L.). Int. J. Mol. Sci..

[8] Kirov I., Dudnikov M., Merkulov P., Shingaliev A., Omarov M., Kolganova E., Sigaeva A., Karlov G., Soloviev A. (2020). Nanopore RNA Sequencing Revealed Long Non-Coding and LTR Retrotransposon-Related RNAs Expressed at Early Stages of Triticale SEED Development. Plants.

[9] Panda K., Slotkin R.K. (2020). Long-Read cDNA Sequencing Enables a “Gene-Like” Transcript Annotation of Transposable Elements. Plant Cell.

[10] Chaparro C., Gayraud T., De Souza R.F., Domingues U.S., Akaffou S., Vanzela A.L.L., De Kochko A., Rigoreau M., Crouzillat M., Hamon S. (2015). Terminal-Repeat Retrotransposons with GAG Domain in Plant Genomes: A New Testimony on the Complex World of Transposable Elements. Genome Biol. Evol..

[11] Kirov I., Merkulov P., Dudnikov M., Polkhovskaya E., Komakhin R.A., Konstantinov Z., Gvaramiya S., Ermolaev A., Kudryavtseva N., Gilyok M. (2021). Transposons Hidden in *Arabidopsis thaliana* Genome Assembly Gaps and Mobilization of Non-Autonomous LTR Retrotransposons Unravelled by Nanotei Pipeline. Plants.

[12] Almeida M.V., Vernaz G., Putman A.L., Miska E.A. (2022). Taming transposable elements in vertebrates: From epigenetic silencing to domestication. Trends Genet..

[13] Wang J., Han G.-Z. (2021). Unearthing LTR Retrotransposon *gag* Genes Co-opted in the Deep Evolution of Eukaryotes. Mol. Biol. Evol..

[14] Cosby R.L., Judd J., Zhang R., Zhong A., Garry N., Pritham E.J., Feschotte C. (2021). Recurrent evolution of vertebrate transcription factors by transposase capture. Science.

[15] Modzelewski A.J., Chong J.G., Wang T., He L. (2022). Mammalian genome innovation through transposon domestication. Nature.

[16] Zhang R.-G., Li G.-Y., Wang X.-L., Dainat J., Wang Z.-X., Ou S., Ma Y. (2022). TEsorter: An accurate and fast method to classify LTR-retrotransposons in plant genomes. Hortic. Res..

[17] Neumann P., Novák P., Hoštáková N., Macas J. (2019). Systematic survey of plant LTR-retrotransposons elucidates phylogenetic relationships of their polyprotein domains and provides a reference for element classification. Mob. DNA.

[18] He L., Huang H., Bradai M., Zhao C., You Y., Ma J., Zhao L., Lozano-Durán R., Zhu J.-K. (2022). DNA methylation-free Arabidopsis reveals crucial roles of DNA methylation in regulating gene expression and development. Nat. Commun..

[19] Baubec T., Pecinka A., Rozhon W., Scheid O.M. (2009). Effective, homogeneous and transient interference with cytosine methylation in plant genomic DNA by zebularine. Plant J..

[20] Boonjing P., Masuta Y., Nozawa K., Kato A., Ito H. (2020). The effect of zebularine on the heat-activated retrotransposon ONSEN in Arabidopsis thaliana and Vigna angularis. Genes Genet. Syst..

[21] Konečná K., Sováková P., Anteková K., Fajkus J., Fojtová M. (2021). Distinct Responses of Arabidopsis Telomeres and Transposable Elements to Zebularine Exposure. Int. J. Mol. Sci..

[22] Thieme M., Lanciano S., Balzergue S., Daccord N., Mirouze M., Bucher E. (2017). Inhibition of RNA polymerase II allows controlled mobilisation of retrotransposons for plant breeding. Genome Biol..

[23] Kirov I., Merkulov P., Gvaramiya S., Komakhin R., Omarov M., Dudnikov M., Kocheshkova A., Konstantinov Z., Solo-viev A., Karlov G. (2021). Illuminating the plant transposon insertion landscape in real time using Cas9-targeted Nanopore sequencing and a novel pipeline. BioRxiv.

[24] de la Rubia I., Srivastava A., Xue W., Indi J.A., Carbonell-Sala S., Lagarde J., Albà M.M., Eyras E. (2022). RATTLE: Reference-free reconstruction and quantification of transcriptomes from Nanopore sequencing. Genome Biol..

[25] Diesh C., Stevens G.J., Xie P., Martinez T.D.J., Hershberg E.A., Leung A., Guo E., Dider S., Zhang J., Bridge C. (2022). JBrowse 2: A modular genome browser with views of synteny and structural variation. BioRxiv.

[26] Llorens C., Futami R., Covelli L., Dominguez-Escriba L., Viu J.M., Tamarit D., Aguilar-Rodríguez J., Vicente-Ripolles M., Fuster G., Bernet G.P. (2010). The Gypsy Database (GyDB) of mobile genetic elements: Release 2.0. Nucleic Acids Res..

[27] Qiu F., Ungerer M.C. (2018). Genomic abundance and transcriptional activity of diverse gypsy and copia long terminal repeat retrotransposons in three wild sunflower species. BMC Plant Biol..

[28] Mascagni F., Vangelisti A., Usai G., Giordani T., Cavallini A., Natali L. (2020). A computational genome-wide analysis of long terminal repeats retrotransposon expression in sunflower roots (*Helianthus annuus* L.). Genetica.

[29] Drost H.-G. (2020). LTRpred: De Novo annotation of intact retrotransposons. J. Open Source Softw..

[30] Ellinghaus D., Kurtz S., Willhoeft U. (2008). LTRharvest, an efficient and flexible software for de novo detection of LTR retrotransposons. BMC Bioinform..

[31] Nunes R.D.C., Orozco-Arias S., Crouzillat D., Mueller L.A., Strickler S.R., Descombes P., Fournier C., Moine D., de Kochko A., Yuyama P.M. (2018). Structure and Distribution of Centromeric Retrotransposons at Diploid and Allotetraploid Coffea Centromeric and Pericentromeric Regions. Front. Plant Sci..

[32] Orozco-Arias S., Liu J., Tabares-Soto R., Ceballos D., Domingues D.S., Garavito A., Ming R., Guyot R. (2018). Inpactor, Integrated and Parallel Analyzer and Classifier of LTR Retrotransposons and Its Application for Pineapple LTR Retrotransposons Diversity and Dynamics. Biology.

[33] Peng H., Mirouze M., Bucher E. (2022). Extrachromosomal circular DNA: A neglected nucleic acid molecule in plants. Curr. Opin. Plant Biol..

[34] Letunic I., Bork P. (2021). Interactive Tree of Life (iTOL) v5: An online tool for phylogenetic tree display and annotation. Nucleic Acids Res..

[35] Delahaye C., Nicolas J. (2021). Sequencing DNA with nanopores: Troubles and biases. PLoS ONE.

[36] Sanderson N., Kapel N., Rodger G., Webster H., Lipworth S., Street T., Peto T., Crook D., Stoesser N. (2022). Compar-ison of R9.4.1/Kit10 and R10/Kit12 Oxford Nanopore flowcells and chemistries in bacterial genome reconstruction. BioRxiv.

[37] Vangelisti A., Mascagni F., Giordani T., Sbrana C., Turrini A., Cavallini A., Giovannetti M., Natali L. (2019). Arbuscular mycorrhizal fungi induce the expression of specific retrotransposons in roots of sunflower (*Helianthus annuus* L.). PLoS ONE.

[38] Baduel P., Quadrana L., Colot V. (2021). Plant Transposable Elements, Methods and Protocols. Methods Mol Biol..

[39] Quadrana L., Bortolini Silveira A., Mayhew G.F., Leblanc C., Martienssen R.A., Jeddeloh J.A., Colot V. (2016). The Arabidopsis thaliana mobilome and its impact at the species level. eLife.

[40] Lanciano S., Mirouze M. (2018). Transposable elements: All mobile, all different, some stress responsive, some adaptive?. Curr. Opin. Genet. Dev..

[41] Quesneville H. (2020). Twenty years of transposable element analysis in the Arabidopsis thaliana genome. Mob. DNA.

[42] Baduel P., Leduque B., Ignace A., Gy I., Gil J., Loudet O., Colot V., Quadrana L. (2021). Genetic and environmental modulation of transposition shapes the evolutionary potential of Arabidopsis thaliana. Genome Biol..

[43] Prochazkova K., Finke A., Tomaštíková E.D., Filo J., Bente H., Dvořák P., Ovečka M., Šamaj J., Pecinka A. (2021). Zebularine induces enzymatic DNA–protein crosslinks in 45S rDNA heterochromatin of Arabidopsis nuclei. Nucleic Acids Res..

[44] Rogers S.O., Bendich A.J. (1985). Extraction of DNA from milligram amounts of fresh, herbarium and mummified plant tissues. Plant Mol. Biol..

[45] Negm S., Greenberg A., Larracuente A.M., Sproul J.S. (2020). RepeatProfiler: A pipeline for visualization and comparative analysis of repetitive DNA profiles. Mol. Ecol. Resour..

[46] Gu Z., Eils R., Schlesner M. (2016). Complex heatmaps reveal patterns and correlations in multidimensional genomic data. Bioinformatics.

[47] Badouin H., Gouzy J., Grassa C.J., Murat F., Staton S.E., Cottret L., Lelandais-Brière C., Owens G.L., Carrère S., Mayjonade B. (2017). The sunflower genome provides insights into oil metabolism, flowering and Asterid evolution. Nature.

[48] Li H. (2018). Minimap2: Pairwise alignment for nucleotide sequences. Bioinformatics.

[49] Li H., Handsaker B., Wysoker A., Fennell T., Ruan J., Homer N., Marth G., Abecasis G., Durbin R. (2009). 1000 Genome Project Data Processing Subgroup. The Sequence Alignment/Map format and SAMtools. Bioinformatics.

[50] Kim D., Paggi J.M., Park C., Bennett C., Salzberg S.L. (2019). Graph-based genome alignment and genotyping with HISAT2 and HISAT-genotype. Nat. Biotechnol..

[51] Liao Y., Smyth G.K., Shi W. (2014). feature Counts: An efficient general purpose program for assigning sequence reads to genomic features. Bioinformatics.

[52] Wickham H. (2011). ggplot2. WIREs Comput. Stat..

