# Supplementary material for: Epigenetic Stress and Long-Read cDNA Sequencing of Sunflower (Helianthus annuus L.) Revealed the Origin of the Plant Retrotranscriptome
Source: Plants (Basel). 2022 Dec 19;11(24):3579. doi: 10.3390/plants11243579 (PMC9784723; doi:10.3390/plants11243579)
Supplement: Supplementary file 1 [file plants-11-03579-s001.zip › plants-2072757-supplementary.pdf]

Supplementary Table S1. Genomic features and encoded retrotransposon domains of 56 expressed RTEs.

Chromosome	Start	End	RATTLE_id	Length	Reads count	Superfamily	Clade	RNA_encodes	Classification
5	111041853	111048190	cluster_10061	2095	21	Copia	Tork	GAG	nonLTR-RTEs
6	15948848	15952707	cluster_4633	1682	18	Copia	TAR	GAG	nonLTR-RTEs
10	10198869	10201567	cluster_7519	1391	49	Gypsy	Retand	GAG	nonLTR-RTEs
10	26092473	26093776	cluster_8141	1362	6	Copia	Ivana	RT-RH	LTR-RTEs
11	115012818	115016428	cluster_399	2950	92	Copia	Ivana	RT-RH	LTR-RTEs
11	189552291	189552871	cluster_24693	1246	57	Copia	Ale	GAG	LTR-RTEs
12	62833938	62881511	cluster_8028	1947	26	Copia	Ivana	RT-RH	LTR-RTEs
16	2368393	2392850	cluster_10355	2030	73	Copia	Ale	GAG	LTR-RTEs
16	163915120	163916719	cluster_14216	1596	13	Copia	Ale	GAG	LTR-RTEs
2	123303689	123304708	cluster_11750	1807	14	Copia	Ale	GAG	LTR-RTEs
2	166928135	166930593	cluster_6420	2414	12	Copia	Ale	GAG	LTR-RTEs
2	167901822	167904173	cluster_6901	3072	146	Copia	Ale	GAG	LTR-RTEs
4	90859498	90859655	cluster_14048	1631	33	Copia	Ale	GAG	LTR-RTEs
7	143032959	143034553	cluster_16611	1484	12	Copia	Ale	GAG	LTR-RTEs
8	14172378	14173905	cluster_18395	1462	33	Copia	Ale	GAG	LTR-RTEs
9	30155070	30156896	cluster_3859	2661	63	Copia	Ivana	RT-RH	LTR-RTEs
15	33119383	33125110	cluster_3551	3792	41	Copia	Ale	GAG-RH-RT	LTR-RTEs
13	58833877	58841753	cluster_5423	2512	25	Gypsy	Tekay	GAG	LTR-RTEs
13	163758439	163761477	cluster_3681	3003	181	Copia	Angela	GAG	LTR-RTEs
15	51651995	51654573	cluster_1380	2310	66	Copia	Ale	GAG	LTR-RTEs
9	154617567	154619891	cluster_7336	2456	80	Gypsy	Galadriel	INT-RT-RH	LTR-RTEs
11	178837568	178838427	cluster_25237	932	19	Copia	Ale	RT	LTR-RTEs
14	32405500	32408839	cluster_6489	2414	42	Copia	Ale	GAG	LTR-RTEs
1	49990110	49991592	cluster_14679	1537	13	Copia	Ale	GAG	nonLTR-RTEs
2	21028153	21029912	cluster_8675	2137	56	Copia	GAG	GAG	nonLTR-RTEs
16	171984474	171986007	cluster_7578	2297	27	Copia	Ale	AP	nonLTR-RTEs
7	40963201	41214845	cluster_23204	1172	113	Gypsy	Tekay	AP	nonLTR-RTEs
1	86032605	86035876	cluster_7965	2138	7	Gypsy	Athila	GAG	nonLTR-RTEs
10	112431674	112433398	cluster_2193	3050	141	Copia	Tork	GAG	nonLTR-RTEs

1	131160102	131160727	cluster_31455	711	11	Copia	Ale	invalidGAG	nonLTR-RTEs
13	117354054	117355489	cluster_15177	1509	58	Copia	Ale	GAG	nonLTR-RTEs
17	88291590	88293112	cluster_17119	1553	22	Copia	Ale	GAG	nonLTR-RTEs
3	123534127	123535158	cluster_6566	2531	153	Copia	SIRE	GAG	nonLTR-RTEs
9	59099804	59101252	cluster_1121	3050	18	Copia	Ale	GAG	nonLTR-RTEs
5	87251873	87252902	cluster_16977	1532	196	Copia	Ale	invalidGAG	nonLTR-RTEs
6	109552656	109553819	cluster_7388	2099	19	Copia	Ale	GAG	nonLTR-RTEs
10	150494674	150497333	cluster_2221	3396	358	Copia	Ale	GAG	nonLTR-RTEs
14	160768694	160771069	cluster_6018	2436	29	Gypsy	Athila	INT-RH	nonLTR-RTEs
10	117711306	117712310	cluster_23187	1123	13	Copia	Ale	RH	nonLTR-RTEs
13	98927461	98928421	cluster_22524	1073	14	Copia	Alesia	RH	nonLTR-RTEs
4	14113081	14114534	cluster_15782	1522	140	Gypsy	Reina	RT	nonLTR-RTEs
6	45198600	45200565	cluster_8568	2126	17	Copia	Ivana	RT-RH	nonLTR-RTEs
13	2243236	2244859	cluster_13124	1601	10	Copia	Ivana	GAG	TR-RTEs
12	10211802	10215117	cluster_10335	1852	8	Gypsy	Tekay	GAG	TR-RTEs
1	42269505	42270084	cluster_12205	1683	12	Copia	Ale	GAG	TR-RTEs
9	154551837	154553104	cluster_8430	2444	30	Copia	Ale	GAG	TR-RTEs
3	133060702	133062271	cluster_4322	3026	138	Gypsy	Tekay	GAG	TR-RTEs
4	9198319	9200044	cluster_10766	1866	20	Copia	Ivana	GAG	TR-RTEs
11	129039178	129042115	cluster_7941	3012	29	Copia	Ale	RH	TR-RTEs
12	128632969	128634187	cluster_6807	1350	9	Copia	Ale	RT	TR-RTEs
2	22602400	22603434	cluster_11455	2097	181	Copia	Bianca	INT-RT	TR-RTEs
8	5635039	5636943	cluster_9729	1969	6	Copia	Ivana	RT-RH	TR-RTEs
11	173696772	173698954	cluster_6221	1777	40	Copia	Ale	GAG	LTR-RTEs
10	30308737	30310622	cluster_11705	1821	9	Copia	Ale	invalidGAG	LTR-RTEs
3	55383993	55393222	cluster_25987	1254	6	Gypsy	Tekay	GAG	LTR-RTEs
12	90097445	90098458	cluster_18084	1317	7	Copia	Ale	INT	LTR-RTEs

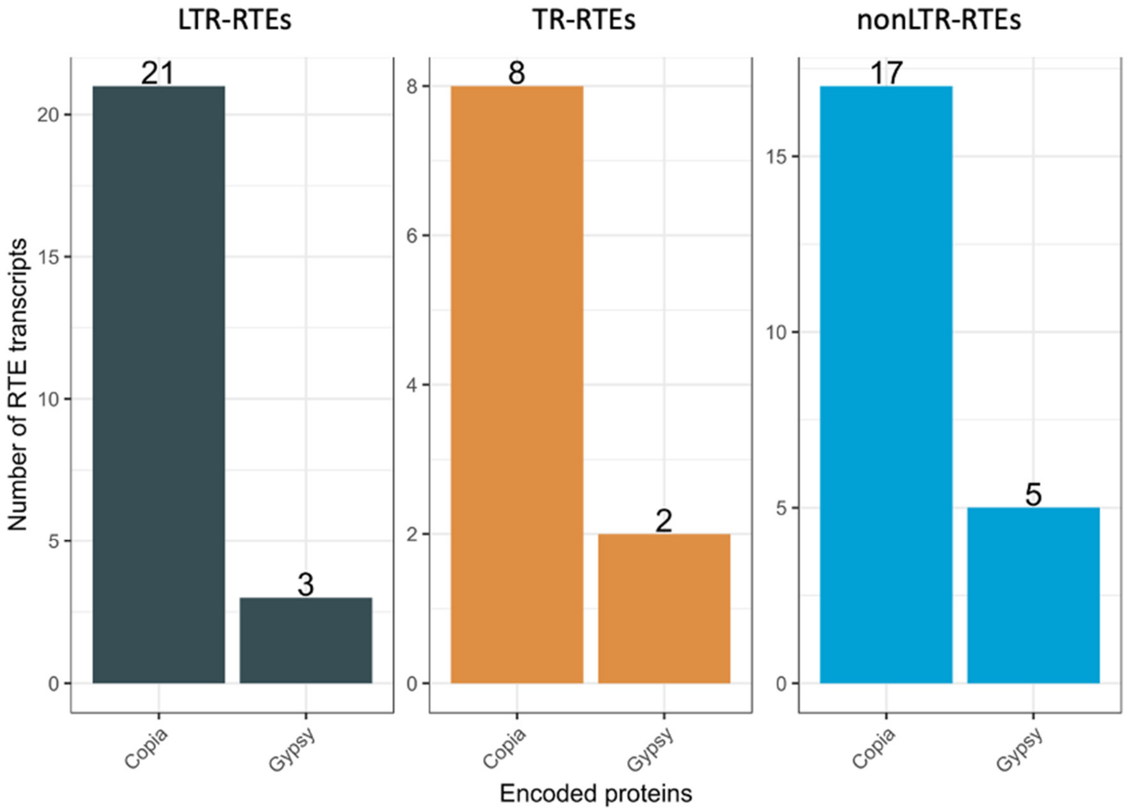

Supplementary Figure S1. Bar plot showing the number of RTE transcripts of different categories expressed from Ty1/Copia and Ty3/Gypsy.

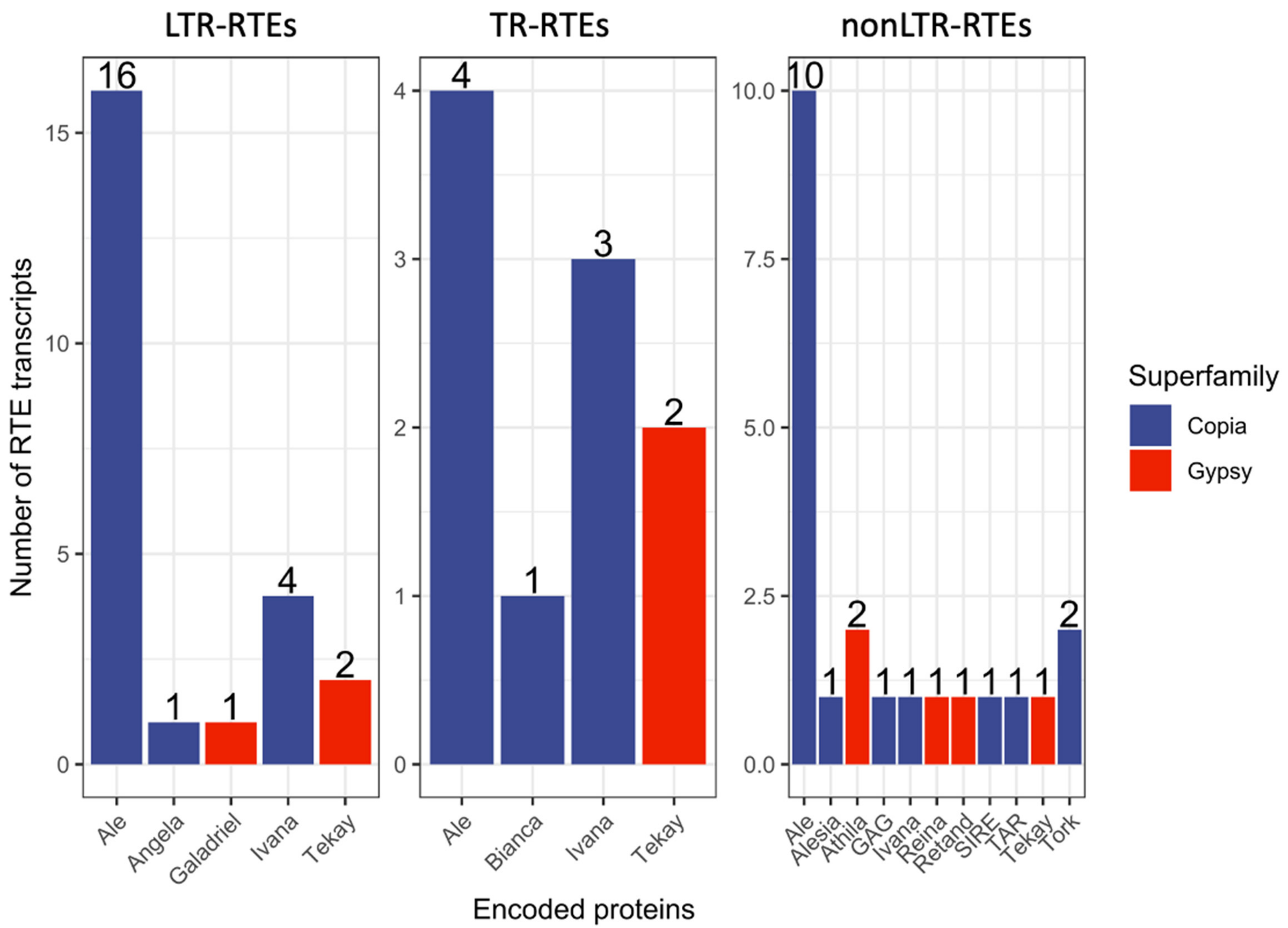

Supplementary Figure S2. Bar plot showing the number of RTE transcripts of different categories expressed from distinct lineages of Ty1/Copia and Ty3/Gypsy.

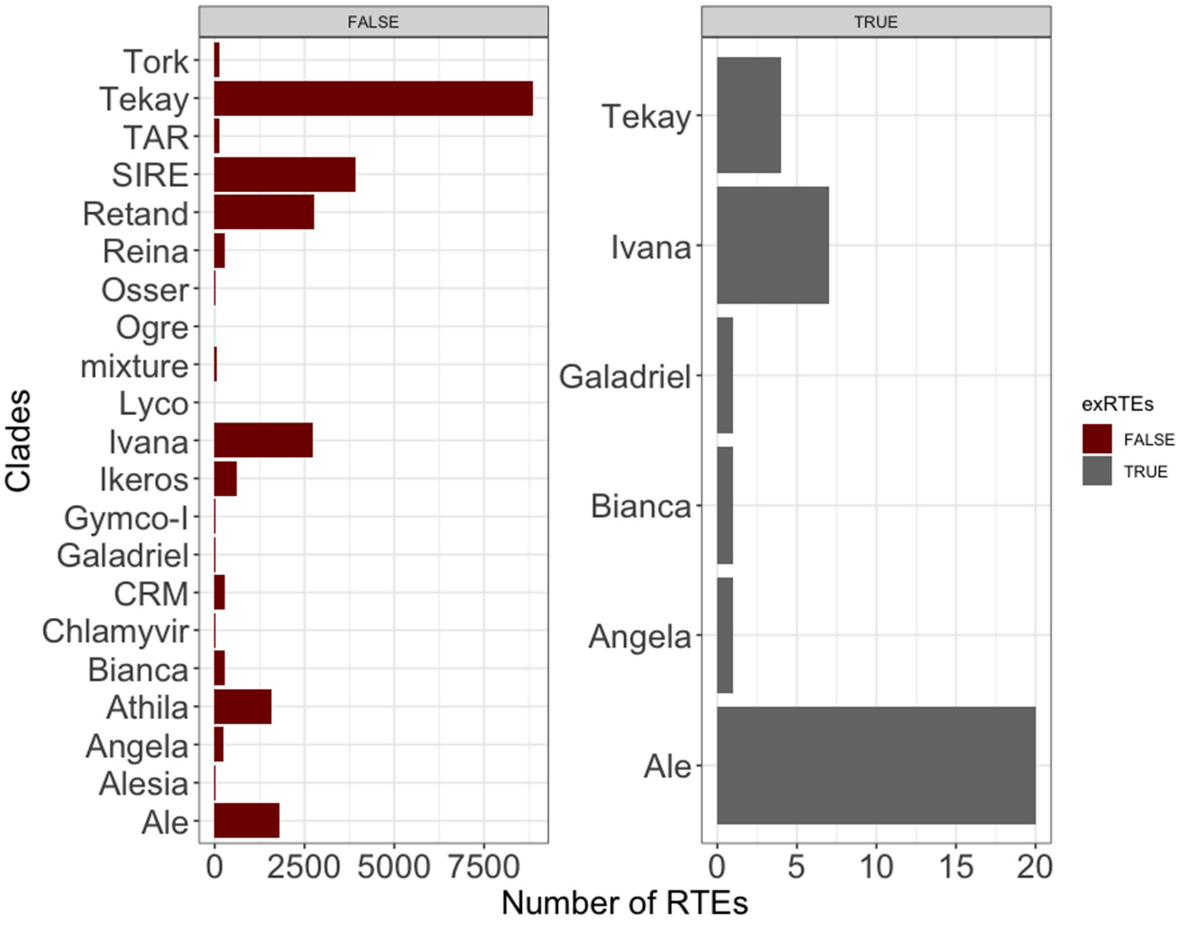

Supplementary Figure S3. Bar plot showing the number of RTEs of different categories expressed from distinct lineages of LTR retrotransposons. Left and right plots correspond to the RTEs identified in the whole genome assembly and expressed RTEs, respectively.

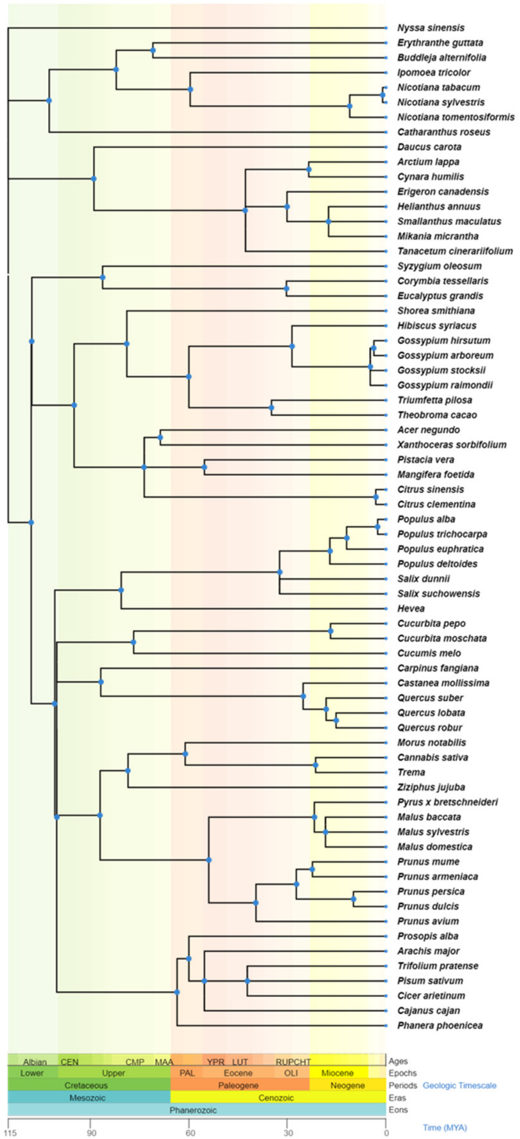

Supplementary Figure S4. Phylogenetic tree of species containing HadGAG2 homologs (TimeTree.org).

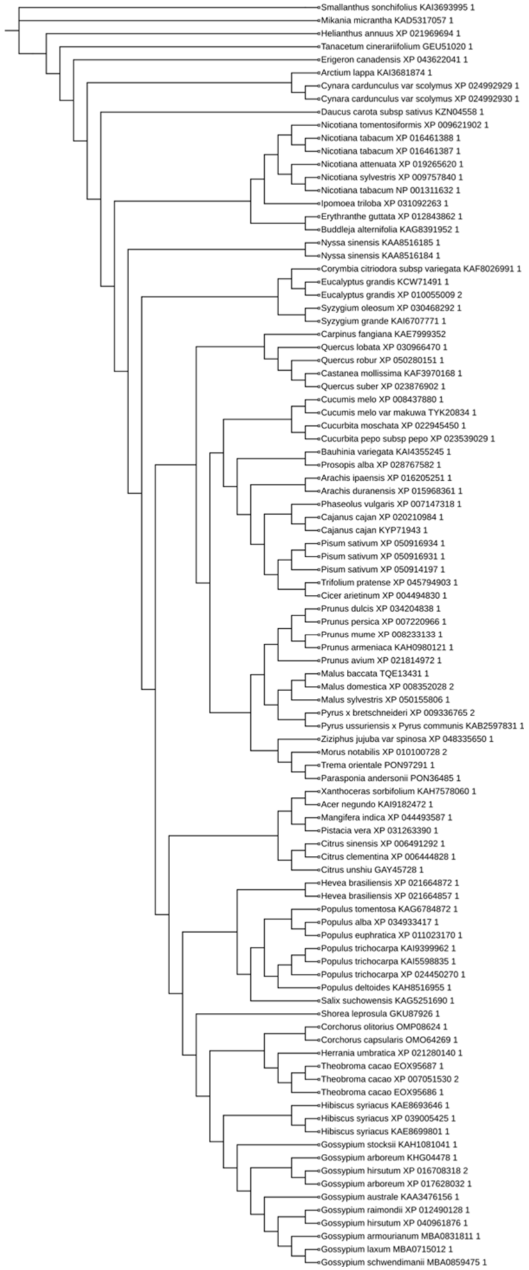

Supplementary Figure S5. Phylogenetic tree based on blastp hits sequences alignment of HadGAG2 homologs (iTOL, NGPhylogeny.fr)
